# The complete mitochondrial genome of a corpse related necrophagous beetle, *Necrodes littoralis* (Coleoptera: Silphidae)

**DOI:** 10.1080/23802359.2021.1914236

**Published:** 2021-04-26

**Authors:** Yangshuai Jiang, Zhuoying Liu, Zhiyun Pi, Qihua Xie, Fanming Meng, Jifeng Cai

**Affiliations:** a Department of Forensic Science, School of Basic Medical Sciences, Central South University, Changsha, China; b Department of Medical Parasitology, School of Basic Medical Sciences, Central South University, Changsha, China

**Keywords:** Mitochondrial genome, *Necrodes littoralis*, Coleoptera, forensic entomology

## Abstract

*Necrodes littoralis* (Linnaeus, 1758) (Coleoptera: Silphidae) is recognized as an important forensically beetle species. In this study, we report the mitogenome of *N. littoralis.* The total length of the mitogenome was 17,830bp (GenBank accession no. MW415274). Two ribosomal RNAs, 13 protein-coding genes, 22 transfer RNAs and a non-coding control region were identified. The base composition of *N. littoralis* was A (39.27%), G (9.49%), T (37.03%), and C (14.21%), respectively. Phylogenetic analysis indicated that *N. littoralis* is closely related to *Diamesus osculans.*

Insects of Coleoptera, also known as beetles, species from the order beetles (e.g., Silphidae, Cleridae and Dermestidae) can provide vital insect evidence for criminal investigations, which play an important role for estimating postmortem intervals (PMI) in decomposed corpses (Vasconcelos and Araujo 2012). *Necrodes littoralis* (Linnaeus, 1758) (Coleoptera: Silphidae) is one of the forensically important beetle (Charabidze et al. 2016). It breeds primarily on large vertebrate carrion by consuming decaying tissues or preying on blowfly larvae (Frątczak and Matuszewski 2014). And it frequently colonizes human carcasses in the later stage of decomposition, especially in forest environments (Matuszewski et al. 2011). In the recent years, *N. littoralis* have been studied for determining PMI, including the calculation of the developmental data of the larvae and pupae (Frątczak and Matuszewski 2014; Novák et al. 2020), but only little genetic information can be available. Therefore, this study provided the complete mitochondrial genome (mitogenome) of *N. littoralis*.

Eight adult specimens of *N. littoralis* were trapped by pig liver on 1st July 2020 from Jilin city (41°14′ N; 125°58′ E), Jilin province, China. All samples were frozen in liquid nitrogen and then stored at −80 °C in Meng’s medical insect herbarium (School of Basic Medical Sciences, Central South University) with a unique number (CSU-KMU-MG20200715-03). According to the manufacture’s instruction, QIANamp Micro DNA Kit (QIANGEN BIOTECH CO., LTD) was used to extract total DNA from an adult specimen of *N. littoralis*. The sequencing was performed on Platform of Illumina HiSeq 2500 (150 bp pared-end). Then the mitogenome of *N. littoralis* was assembled by MITObim software (Christoph et al. 2013). All genes were annotated by the MITOS2 Web Server (http://mitos2.bioinf.unileipzig.de/index.py) under the invertebrate mitochondrial code (Bernt et al. 2013).

In our study, the mitogenome of *N. littoralis* was 17,830 bp in size (GenBank accession no. MW415274.1), containing two ribosomal RNA (rRNA) genes, 13 protein-coding genes (PCGs), 22 transfer RNA (tRNA) genes and a non-coding control region. The composition of genes, especially the PCGs, is roughly coincide with the arrangement of ancestral metazoan (Cameron 2014). The base composition of *N. littoralis* was A (39.27%), G (9.49%), T (37.03%), and C (14.21%), respectively. Furthermore, mitogenome has been recognized as an important molecular marker for evolution analysis (Nie and Yang 2014). Phylogeny tree of *N. littoralis* was conducted with eight Coleoptera species based on 13 PCGs by maximum likelihood (ML) method implemented in IQ-TREE v.1.6.8 (Lam-Tung et al. 2015), and two species of flesh flies (Diptera: Sarcophagidae) were used as an outgroup (Figure 1). The result showed that *N. littoralis* is closely related to *Diamesus osculans* with high support value. This mitogenome data provide valuable resource for further exploring the evolutionary relationship within Coleoptera.

**Figure 1.**
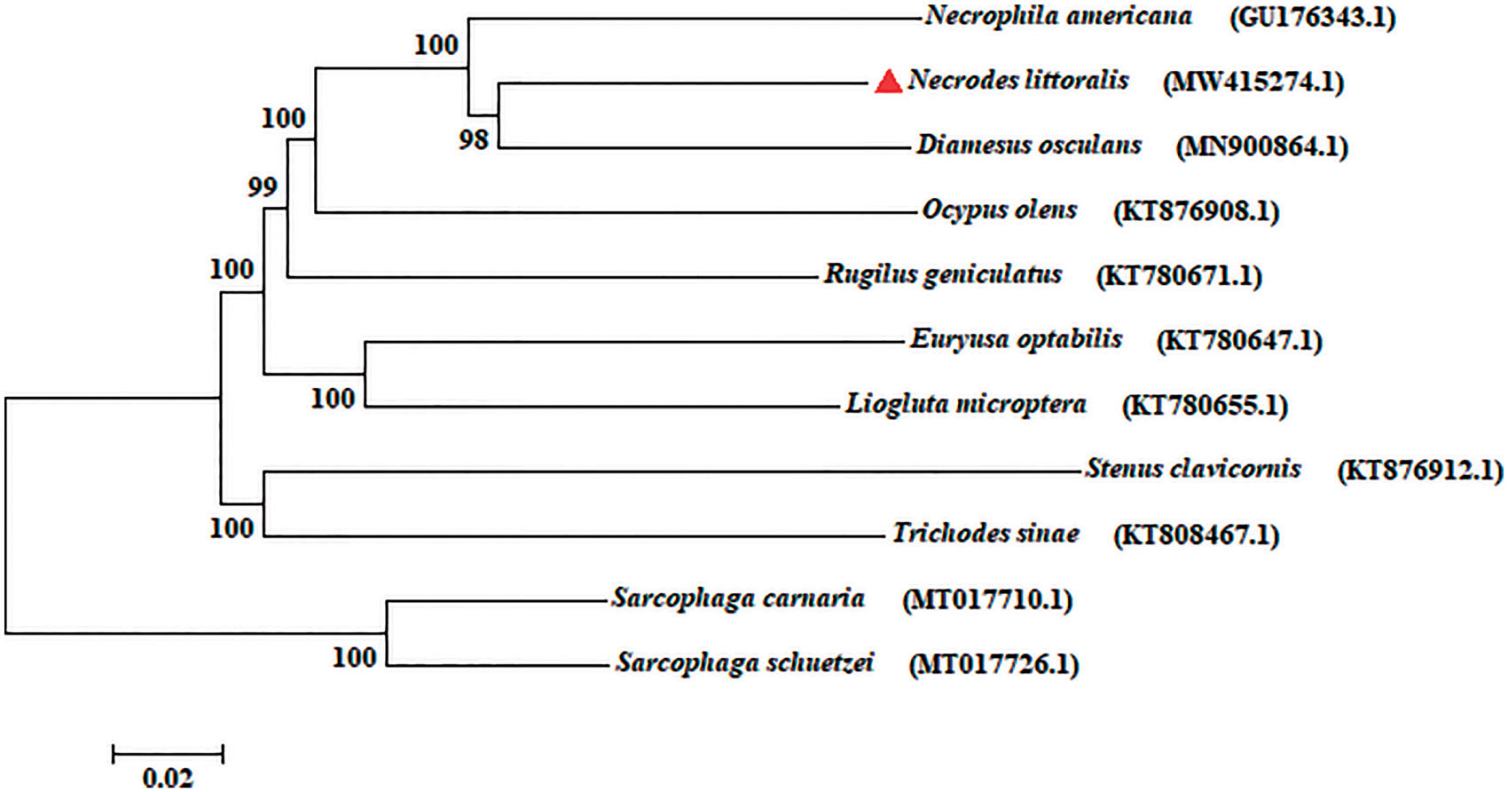
Phylogeny tree of *N. littoralis* was constructed with eight Coleoptera species based on 13 PCGs by maximum likelihood (ML) method, and two species of flesh flies (Diptera: Sarcophagidae) were used as an outgroup.

## Data Availability

The *Necrodes littoralis* complete mitochondrial genome sequence data is available at NCBI:MW415274.1. The accession numbers of BioProject, SRA, and BioSample were been published in NCBI: PRJNA690600 (https://www.ncbi.nlm.nih.gov/bioproject/PRJNA690600), SRR13385770 (https://www.ncbi.nlm.nih.gov/sra/SRR13385770) and SAMN17255111 (https://www.ncbi.nlm.nih.gov/biosample/SAMN17255111/), respectively. All samples were stored in Meng’s lab (Fanming Meng Ph.D, mengfanming1984@163.com).
